# SEX DIFFERENCE IN EARLY FRAILTY TRANSITIONS, HEALTHCARE USE, AND MEDICARE PAYMENT IN OLDER MEXICAN AMERICANS

**DOI:** 10.1093/geroni/igz038.1837

**Published:** 2019-11-08

**Authors:** Chih-Ying Cynthia Li, Amol Karmarkar, Lin-Na Chou, Soham Al Snih, Yong-Fang Kuo, Kenneth Ottenbacher

**Affiliations:** 1 University of Texas Medical Branch, Galveston, Texas, United States; 2 University of Texas Medical Branch at Galveston, Galveston, Texas, United States

## Abstract

This study investigated sex difference in early frailty transitions on one-year follow-up healthcare utilization and Medicare payment. We used the linked Medicare claims data and the Hispanic Established Populations for the Epidemiological Study of the Elderly (Hispanic-EPESE) survey, using longitudinal analyses for 789 older Mexican Americans ≥70 years old in 1998/99. Participants were divided into five transition groups: 1) remain non-frail, 2) improve (pre-frail to non-frail, frail to non-frail, frail to pre-frail), 3) remained pre-frail, 4) remained frail, 5) worse (non-frail to pre-frail, non-frail to frail, pre-frail to frail) based on their frailty status between Wave 3 (1998/99) and Wave 4 (2000/01). Main outcomes were: (a) healthcare utilization (hospitalization, emergency room admission, physician visit) and (b) Medicare payment (total and outpatient payments) from 2000/01 to 12 months after. Mean age was 78.8 (SD=5.1) and 60.3% were female in 1998/99. We found sex had significant interaction effects on one-year follow-up hospitalization and Medicare outpatient payment. Compared to the remained no-frail group, males who remained pre-frail (Odds Ratio [OR]= 3.62, 95% CI=1.18-11.2), remained frail (OR= 7.59. 95% CI= 1.74-33.1) and worse (OR=4.54, CI=1.74-11.8) had higher risk for hospitalization. Males in the worse group also had significantly higher Medicare outpatient payment (OR=2.58, CI=1.46-4.56). Same associations were not observed in females. However, both genders used similar frequency and type of outpatient services, as the top services were evaluation and management services. Our results suggested research is needed to examine balance between sex differences, frailty improvements, resources needed and total care expenditure in this population.

